# *De Novo* Assembly and Transcriptome Analysis of the Mediterranean Fruit Fly *Ceratitis capitata* Early Embryos

**DOI:** 10.1371/journal.pone.0114191

**Published:** 2014-12-04

**Authors:** Marco Salvemini, Kallare P. Arunkumar, Javaregowda Nagaraju, Remo Sanges, Valeria Petrella, Archana Tomar, Hongyu Zhang, Weiwei Zheng, Giuseppe Saccone

**Affiliations:** 1 Department of Biology, University of Naples Federico II, Naples, Italy; 2 Centre for DNA Fingerprinting and Diagnostics, Hyderabad, India; 3 Stazione Zoologica “Anton Dohrn”, Naples, Italy; 4 State Key Laboratory of Agricultural Microbiology and Institute of Urban and Horticultural Pests, College of Plant Science and Technology, Huazhong Agricultural University, Wuhan, People’s Republic of China; Universidade Federal do Rio de Janeiro, Brazil

## Abstract

The agricultural pest *Ceratitis capitata,* also known as the Mediterranean fruit fly or Medfly, belongs to the Tephritidae family, which includes a large number of other damaging pest species. The Medfly has been the first non-drosophilid fly species which has been genetically transformed paving the way for designing genetic-based pest control strategies. Furthermore, it is an experimentally tractable model, in which transient and transgene-mediated RNAi have been successfully used. We applied Illumina sequencing to total RNA preparations of 8–10 hours old embryos of *C. capitata,* This developmental window corresponds to the blastoderm cellularization stage. In summary, we assembled 42,614 transcripts which cluster in 26,319 unique transcripts of which 11,045 correspond to protein coding genes; we identified several hundreds of long ncRNAs; we found an enrichment of transcripts encoding RNA binding proteins among the highly expressed transcripts, such as CcTRA-2, known to be necessary to establish and, most likely, to maintain female sex of *C. capitata*. Our study is the first *de novo* assembly performed for *Ceratitis capitata* based on Illumina NGS technology during embryogenesis and it adds novel data to the previously published *C. capitata* EST databases. We expect that it will be useful for a variety of applications such as gene cloning and phylogenetic analyses, as well as to advance genetic research and biotechnological applications in the Medfly and other related Tephritidae.

## Introduction

*Ceratitis capitata*, the Mediterranean fruit fly (Medfly) is an agricultural pest insect of high economic importance. During the last centuries, this pest spread from Africa to other continents, and it is continuing to move and colonize new geographic areas such as more recently in China [1]–[4]. Because of its wide host range (more than 200 vegetable species), its prodigious reproductive capacity, and its surprising adaptability to new and even adverse ecological environments the Medfly is one of the world’s most destructive fruit pests, with the potential to cause up to billions of Euros losses within a few months at the national level [5], [6]. In contrast to other fruit flies, such as *Drosophila melanogaster*, which lays eggs on decaying fruit, *C. capitata* causes damage to fruit crops by laying eggs inside intact fruit [7].

The genetic knowledge gained from studies using the model insect *Drosophila*, was an important premise to develop genetic based methods for sexing males in *C. capitata* in pest control programs such as the Sterile Insect Technique (SIT) [8] and the Release of Insects carrying Dominant conditional Lethal genes (RIDL) [9]–[11]. In the last two decades, a comparative study based on the identification of candidate genes led to the identification of key *C. capitata* sex determining genes. This knowledge has been exploited to obtain male-only progeny by suppressing the female determining gene *Cctransformer* (or *Cctransformer-2*) by RNAi or by conditional lethality setting the stage for the insect biotechnology to control pest and disease vector insects in the field [8], [12]–[20]. Indeed, the private sector also moved into this field to develop *C. capitata* transgenic strains by introducing a conditional female-specific dominant lethal construct, [14]. Biotechnology based control strategies have been developed in pest and in animal disease vectors and some have been tested in the field [21], [22] and many more are expected to be realized in the near future [23]–[26].

Various differential expression techniques have been used to identify *C. capitata* genes which are potentially involved in reproduction, mating behaviour [27], in sexual maturation [5], in mating, post mating [28] and in olfaction [29]. Similar studies have been started recently in other related Tephritidae, such as *Anastrepha suspensa* and *Bactrocera dorsalis* [30], [31]. Other investigators applied molecular subtractive strategies isolating *C. capitata* genes either specifically expressed during the cellularization stage or which are male-specific [32; Salvemini *et al*., manuscript submitted]. Furthermore, gene promoters which are active in oogenesis and in early embryos have been identified both in *C. capitata* and in the disease vector *Aedes aegypti*. These regulatory regions can be used to construct transgenes which express maternal or early embryonic products [33], [34].

The cost- and time-effectiveness of Next Generation Sequencing technologies [35], [36] and the development of new reverse genetics tools (RNAi, TALENs and CRISPR-Cas9) allow many insect species to become model organisms in evolutionary and developmental studies as well as substrates for biotechnological applications [37]–[43]. Integrated international initiatives recently started to focus on comparative genomics studies in insects, which eventually will be very useful to develop alternative control strategies for pest species or disease vectors as well as to improve the existing ones [26], [44]–[49].

Detailed knowledge of stage-specific and tissues-specific transcription in *C. capitata* is, however desirable to broaden the range of biotechnical techniques which can be used for its control. For example, Medfly male development is based on switching OFF the *transformer* gene during embryogenesis. This masculinising event can be artificially induced in karyotypically females by RNAi based silencing of *tra* (or of its co-regulator *tra-2*) [15], [16], [50]–[54]. Previous studies have revealed that sex determination starts around blastoderm cellularization in *C. capitata* [15], [55], at the same stage as determined in other insect species, such as the dipterans, *D. melanogaster* [56] and *Musca domestica* [53], the lepidopteran *Bombyx mori* [57], the coleopteran *Tribolium castaneum* [58], the hymenopterans *Apis mellifera* [59] and *Nasonia vitripennis* [60], [61].

In this study we used, for the first time, Illumina short read sequencing technology for transcriptome analysis of mixed male and female embryos aged between 8–10 hours after egg lay), the stage at which sex becomes irreversibly fixed. Nearly 21 million 100-bp paired-end (PE) reads were used to assemble 42,614 transcripts, which clustered into 26,319 unigenes (MEET database; Medfly Early Embryonic Transcripts). Comparison with the previous Medfly EST database revealed that 78% of the assembled unigenes (20,400) were newly identified *C. capitata* transcripts. The MEET database and the Medfly genome and transcriptome data, which soon should be published by the Medfly International Consortium (Handler, AM:, pers. Comm.), will help to apply *in silico* approaches such as chromosome quotient or *in silico* subtraction to identify Y-linked genes, male-specific or male-biased genes and to search for the primary signal of the sex determination of Medfly, the Male Determining Factor (M-factor) [57], [62]–[64].

## Results and Discussion

### Sequencing and *de novo* assembly

**Figure 1:**
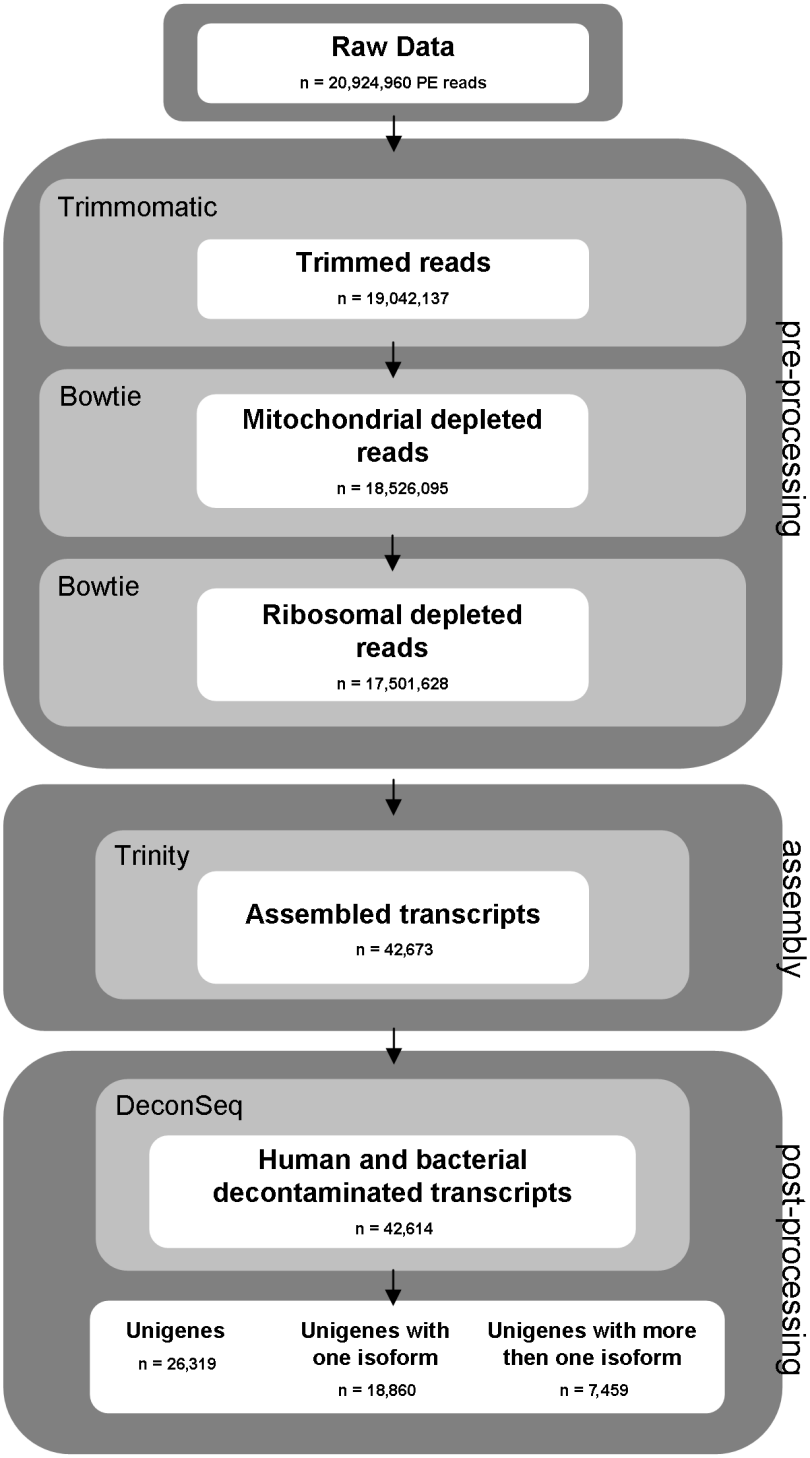
Filtering and assembling pipeline flowchart. Data files are represented as white boxes. Software utilized for each step are represented as light grey boxes while dark grey boxes represent the phases of the filtering and assembling pipeline.

A cDNA library for paired-end (PE) Illumina sequencing was prepared from total RNA isolated from 8–10 hours after egg laying (AEL) embryos. Using an Illumina Genome Analyzer IIx platform, a total of 21 million (M) 100 bp-long PE reads with high sequence quality (Phred score > = 33) were obtained. Figure 1 shows an overview of the sequence analysis workflow of this study. *De novo* assembly of the Illumina reads was performed using Trinity, a de Bruijn graph based assembler which can reconstruct full-length transcripts and corresponding isoforms, without the need of a reference genome [65], [66]. As Trinity algorithm discards low coverage k-mers, no quality trimming of the reads is usually required [65]. However, we assessed if an adapter contamination removal and a quality control of raw reads were performed with three filtering software (NGS-QC-Toolkit, Trimmomatic and Galaxy) [67]–[69], this could improve the overall quality of the Trinity assembly. We compared the performance of these applications under four different conditions and found that Trimmomatic software was the most efficient (see Methods and Methods S1 for further details).

Reads derived from *C. capitata* mitochondrial genome and five ribosomal transcripts (2.71% and 5.38% of the total reads, respectively) were identified by BOWTIE [70] and discarded. The Trinity assembler reconstructed 42,673 transcripts, using a total of 48,815,521 nucleotides, which showed sequence length ranging between 200 bp and 10,000 bp, (average length = 1145 bp) and high N50 value (2,158). The number of assembled transcripts derived from bacterial, viral and human genomes (contamination) was negligible (0.14%) with only 56 transcripts identified by DeconSeq web-tool and discarded [71]. An additional BLAST analysis against the UniVec database found only three transcripts containing 34 bp long Illumina adapters. The final resulting 42,614 transcripts have been listed in the MEET dataset (Medfly Early Embryonic Transcriptome). Sequencing and assembly statistics are summarized in Table 1.

**Table 1:** Sequencing and assembly statistics.

**Number of input paired end reads**	20924960
**Low quality reads**	1882823
**Final HQ reads**	19042137
**% of Final HQ reads**	91
**Mitochondrial and ribosomal depleted reads**	17501628
**Number of assembled transcripts**	42673
**Number of cleaned assembled transcripts**	42614
**Number of assembled nt**	48815521
**N50**	2158
**Average transcript lenght**	1145
**Number of transcripts>1 Kb**	16023
**Number of transcripts>2 Kb**	8136
**Number of transcripts>3 Kb**	3693
**Number of transcripts>5 Kb**	631
**Number of transcripts>10 Kb**	14
**Max transcript lenght**	10406

We selected the longest transcript for each Trinity component and obtained 26,319 clusters of which 18,860 (71.7%) have only 1 isoform while the remaining 7,459 (28.3%) have 2 or more isoforms. Accordingly to other authors, we named these clusters as “unigenes”. The unigenes showed an average length of 764 bp and the N50 value of 1,517; 5,436 (∼25%) reconstructed unigenes were larger than 1,000 bp. In comparison with other similar transcriptome studies [30], [31], [38], [48], the high N50 value and the number of assembled unigenes indicated that both the mRNA-seq and the assembly procedures were very effective. All sequencing reads were deposited into the Short Read Archive (SRA) of the NCBI (Accession Number: SRR1380982). The assembled MEET dataset is freely available at meetbase.evosexdevo.eu, following online registration.

### Coverage assessment

**Figure 2:**
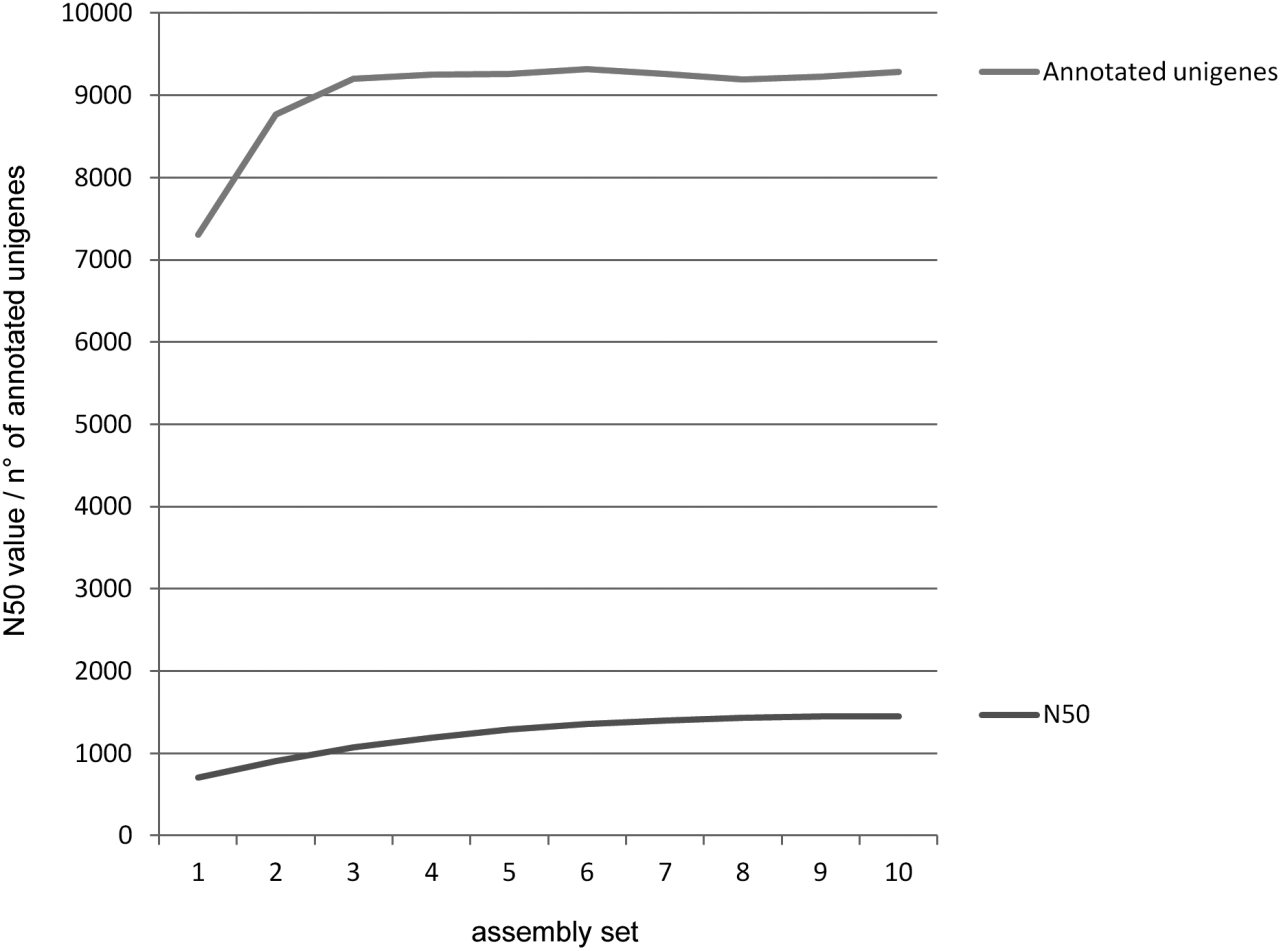
Coverage assessment of the MEET data set. The number of assembly obtained with a progressively large sub-samples of randomly selected raw reads (2****M for each step) is plotted against the number of annotated unigenes by FastAnnotator software (using NCBI nr database) and the N50 value. The number of annotated unigenes reached a plateau after about 8****M reads (green line) while the N50 unigene value continued to increase approximately linearly over the same range (red line).

A transcriptome analysis is effective if the number of assembled transcripts and their lengths approximates the real values. To evaluate how thoroughly our RNA sequencing captured the real diversity of early embryonic Medfly transcripts, we assembled ten independent and progressively larger (2****M for each step) sub-samples of randomly selected raw reads [37]. We counted the total number of unigenes annotated for each assembly using FastAnnotator [72] an automated web-based service, which integrates well-established annotation tools as Blast2GO, PRIAM and RPS BLAST to assign Gene Ontology (GO) terms, Enzyme Commission numbers (EC numbers) and functional domains to query sequences, respectively. The number of unigenes slowly plateaus after ∼8 M reads out of 21****M (Figure 2 - green line). The N50 unigene length increases roughly linearly until ∼12****M when started to plateau, although very slowly (Figure 2 - red line). These results suggest that the 21****M reads is likely sufficient to represent most of the real complexity of embryonic transcriptome present between 8–10 hours AEL. However, we expect that additional mRNA sequencing and *de novo* assembly will slightly lengthen the unigene sequences, mainly in the UTR regions, because novel reads will cover the entire length of the purified and sequenced mRNA, especially for those genes having a lower expression.

To address how closely the assembled unigenes are to the real full-length transcripts, as a first approach, we performed gene-by-gene analyses on a small sample. For size comparison, we randomly selected 27 *C. capitata* cDNA sequences from GenBank, annotated as complete sequence, and compared them to the corresponding transcripts from our MEET dataset (see Table S1). Eight out of twenty-seven MEET transcripts showed the same length, six out of twenty-seven MEET transcripts were longer in size, extending by an average of 665 bp (range of 127–1,884 bp) (see Table S1). Thirteen out of twenty-seven transcripts were not found in the MEET dataset. These genes may not be expressed at the early embryonic stage, such as *ceratotoxin A* [73], *farnesoic acid O-methyltransferase* [74] and *alpha-L-fucosidase* [75]. These 13 mRNAs are indicated with an asterisk in Table S1.

As a second approach, we used the “ortholog hit ratio” (OHR), which provides an estimate of the length of the assembled transcripts relative to a reference genome by comparing only the coding regions [76]. We compared the number of bases in the hit region of *C. capitata* unigenes to the length of the best TBLASTX match with *Drosophila* transcripts, which correspond to orthologous coding regions. The idea of this approach is to test whether the coding regions of the unigene and the reference gene have the same length, An “OHR” value of 1 suggests that a transcript has been assembled to its correct size, while values lower than 1 suggest incomplete assembly (e.g. artificial deletions in the coding region as a result of low coverage).

To calculate the MEET “OHR” values we first identified the 1-to-1 orthologs comparing our data set and the *D. melanogaster* transcriptome (BDGP v5.22). We utilized a custom Perl script to perform a best reciprocal TBLASTX analysis resulting in 2,231 selected MEET unigenes. We observed that half of the selected unigenes cover at least 50% of corresponding *Drosophila* coding regions (average “OHR” and median “OHR” of 0.52 and 0.49, respectively – see Table S2). Similar values have been reported from transcriptome analyses of other insects [37], [76] indicating that the MEET assembly has a high length coverage.

Also, we compared transcript sequences with 5′ and 3′ untranslated regions. We performed BLASTN using the full length of the 2,231 MEET transcripts as queries and the full-length of their *Drosophila* orthologs as target subject sequences. We defined a “full-length hit ratio” metric (“FLHR”) as query length divided by subject length and we observed an average and median values both of 1.04, with about 60% of the 2,231 unigenes having a length equal or higher than the *Drosophila* orthologs (see Table S2). This observation is coherent with the previous OHR, indicating that the assembly was relatively efficient. In addition, for those genes coding for highly conserved proteins, the entire length of the transcript seems to be conserved during evolution even in distantly related species such as *C. capitata* and *Drosophila* (120 million of years, mya; [13], [77].

### Comparisons with available medfly ESTs

Gomulski et al., (2008) [27] and Scolari et al., (2012) [28] have previously assembled 27,167 high-quality ESTs into a total of 15,229 *C. capitata* ESTs from 0–36 hours old embryos, dissected adult heads and testes and male accessory glands. To compare the MEET dataset to other publicly available medfly transcript data sets, all *C. capitata* ESTs deposited at dbEST/GenBank were downloaded (27,167 ESTs with an N50 of 740 bp, 2013.09.01). Since the assembled ESTs described in both papers were not deposited in public databases, we performed, as a first step in our comparative analysis, an assembly of the ESTs dataset using iAssembler software [78], obtaining comparable assembly results with respect to the results of the original papers (see Table S3).

We performed bidirectional BLASTN analyses of the Global EST dataset (13538 assembled ESTs from all tissues and stages) and embryonic EST sub-dataset (5754 assembled ESTs) with the novel MEET unigene dataset (26319 assembled unigenes 8–10 AEL) (Table 2), with a stringent cut-off value of 1e-100, a minimum coverage of the BLAST hit of 30% and a minimum of identity of the BLAST hit of 80%. It is important to notice that the Global EST dataset (and the embryonic EST sub-dataset) includes embryonic transcripts expressed between 0 and 36 hours, which is a much larger time range than the 8–10 hour window of the MEET dataset. Hence, we expected to find a higher complexity in this dataset than in the MEET dataset. Indeed, only 53% Global EST dataset and 83% of Embryonic EST sub-dataset had BLASTN hits to the MEET unigene dataset. Hence 47% of Global EST dataset (6,300 out of 13,538) and 17% of the Embryonic EST sub-dataset (1,004 out of 5,754) is absent in the MEET unigene dataset. The missing transcripts are likely to be expressed only at later stages of development. Interestingly, only 22% of the MEET unigenes had hits to the Global EST dataset. We concluded that 78% unigenes of the MEET unigene dataset (20,447) correspond to newly identified Medfly transcripts, an higher effectiveness of transcriptome analyses when performed by this novel RNA-seq *de novo* assembly approach. Interestingly, only 16% of the MEET unigenes had BLASTN hits to embryonic EST sub-dataset. Hence, 84% of the MEET unigenes (22,136) are new.

**Table 2:** Comparison of the present *C. capitata* MEET dataset with the available *C. capitata* ESTs.

e-value (1E-100) mincoverage (30%) minidentity (80%)	Global ESTs datasetversus MEETunigene dataset	MEET unigenedataset versusGlobal ESTs dataset	Embryonic ESTssub-dataset versusMEET unigene dataset	MEET unigenedataset versusEmbryonic ESTssub-dataset
BLASTN hits	7155	5872	4750	4183
total	13538	26319	5754	26319
%	53	22	83	16
average length of hits	861	1639	802	1796
N50 of hits	828	2411	784	2480

### Functional annotation

We annotated the MEET unigene dataset using FastAnnotator (Chen et al., 2012) obtaining 11,045 unigenes (42% of total 26,319 assembled unigenes) with a significant similarity (E-value<1e^−5^) to proteins present in the NCBI non-redundant (nr) database. Of these 9,098 unigenes (82%) had at least one functional annotation. In particular, 7,948 (87%) mapped to GO terms - level 2, by Blast2Go, 705 (8%) were identified to have at least one enzyme hit in the ENZYME database (http://enzyme.expasy.org/) and 5,965 (65%) had at least one domain, with domain coverage >50%, in PFAM database (http://pfam.sanger.ac.uk/). The 15,274 unigenes without annotation have an N50 value of only 398 bp, a very low value respect to the N50 of the 11,045 unigenes corresponding to putative protein coding genes (N50 = 2231 bp). These non-annotated transcripts could represent 1) non-coding genes having short transcripts and/or 2) non fully assembled 5′ and 3′ UTR regions of very low expressed genes. In the first case, it would be of interest to further investigate the nature and the possible function of this group of short transcripts, enlarging the transcriptome complexity. In the second case, the unigenes could correspond to genes already contained in the 11,045 annotated unigenes, without contributing to transcriptome complexity.

**Figure 3:**
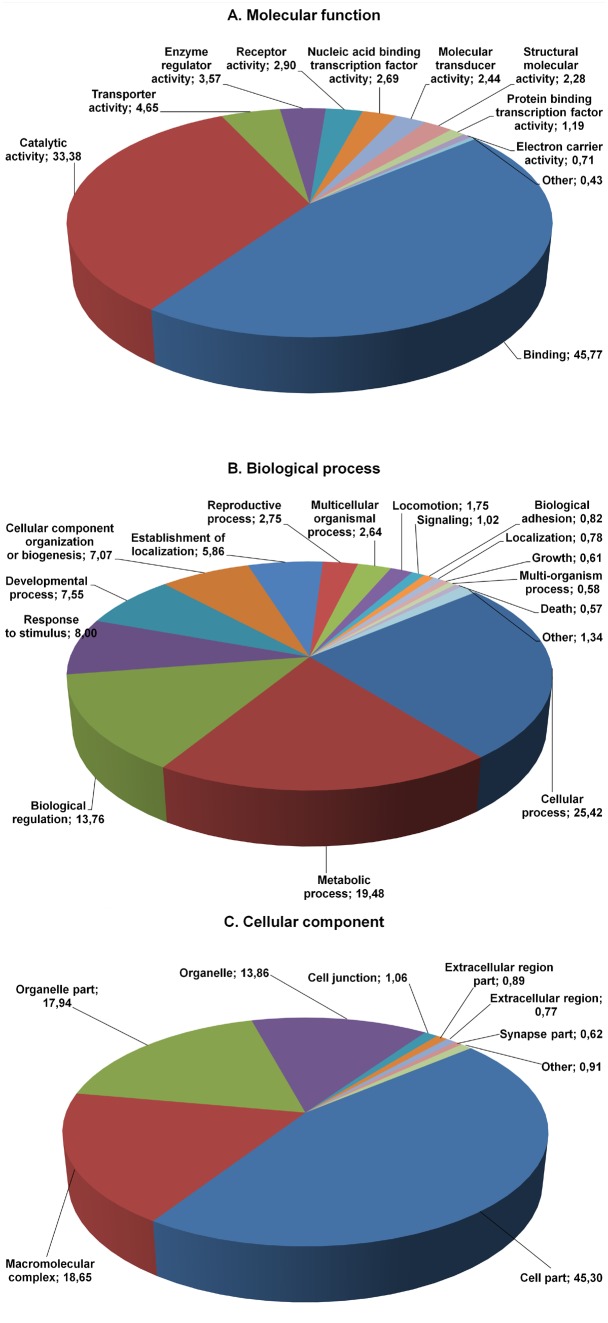
Gene ontology distribution of the MEET data set. GO term distribution, level 2, of our data set derived from the blast2go analysis of the FastAnnotator annotation pipeline. Percentages are indicated after each GO term.

Among the GO 7,948 unigenes, a large diversity of functional categories was observed similar to those found in other dipteran insect transcriptomes. These unigenes were categorized into 53 main function groups of Gene Ontology database, belonging to molecular function, biological process and cellular functional component categories (Figure 3). In the molecular function classification, the most represented classes are “binding” and “catalytic activity” classifications, which contain respectively 45.8% and 33.4% of all unigenes. In the biological process classification the largest classes are “cellular process” (25.4%), “metabolic process” (19.5%) and “biological regulation” (13.8%). In the cellular component categories, “cell” and “organelle” result the most represented (45.3% and 31.8%, respectively).

**Figure 4:**
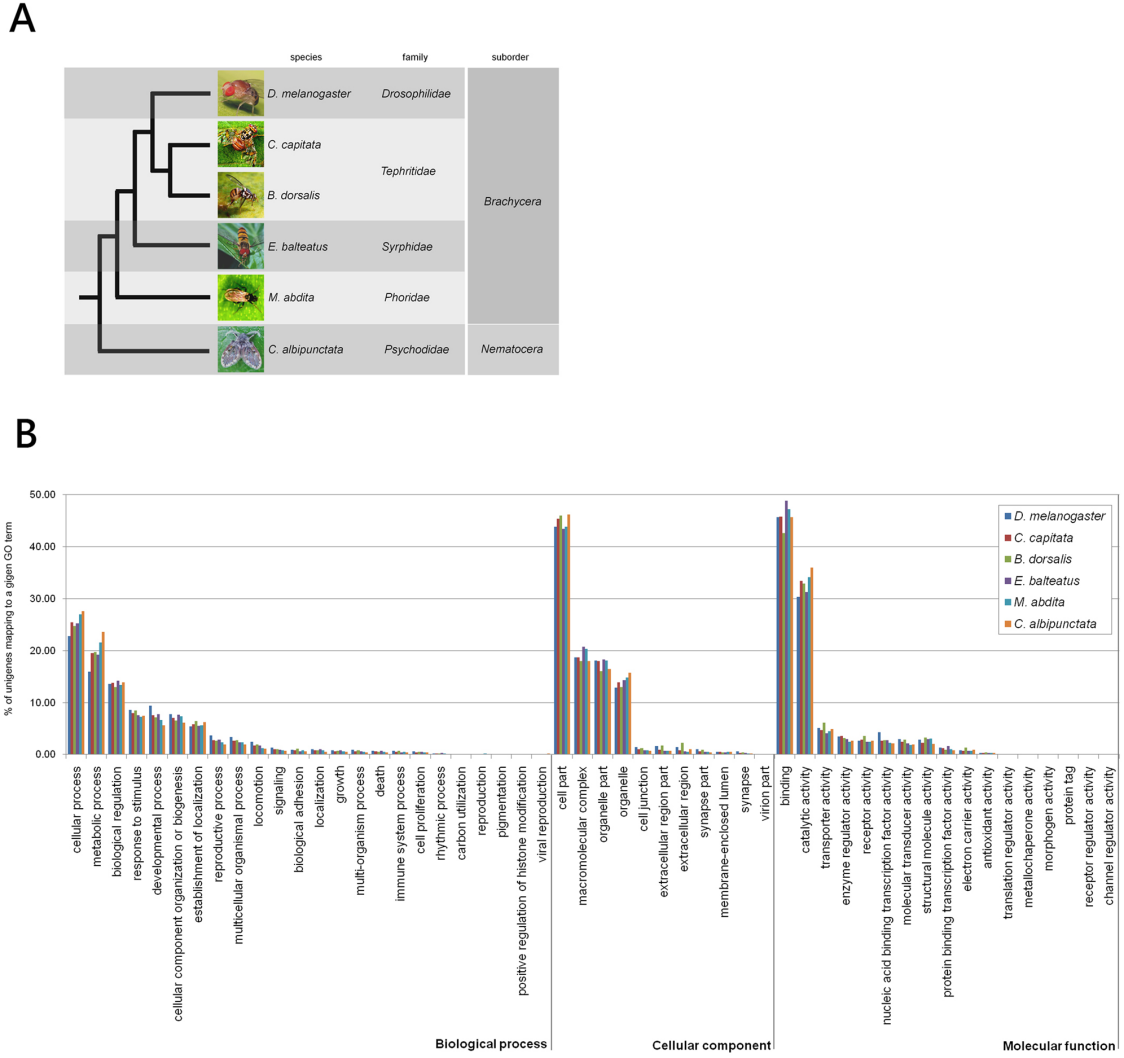
Comparative analysis of GO term distribution in dipteran embryonic transcriptome. (A) A schematic phylogenetic tree of the species included in the comparison of GO term distribution. Families and sub-orders are indicated. The analyzed species span approximately 200 million of years of evolution of the order Diptera [38], [102]. (B) Column heights represent the percentage of the annotated unigene in each assembly assigned to a give GO term, level 2. The relative percentage of unigenes included in each GO categories are comparable among all the analysed species.

We further performed a comparative analysis of the MEET GO terms distribution with the GO terms obtained from available dipteran high-throughput embryonic transcriptome data of *D. melanogaster* (2–16****hrs old embryos) [79], of the Tephritidae species *B. dorsalis* (0–24****hrs old embryos) [80] and of three emerging dipteran experimental model systems: the moth midge *Clogmia albipunctata* (family: Psychodidae) (8–12****hrs old embryos), the scuttle fly *Megaselia abdita* (family: Phoridae) (0–4****hrs old embryos) [38], and the hoverfly *Episyrphus balteatus* (family: Syrphidae) (3–6****hrs old embryos) [81]. These species span about 200 million of years of evolution in the Diptera order (Figure 4A). To obtain comparable datasets, raw data files for each species were downloaded and assembled by Trinity or by iAssembler software followed by a functional annotation step with FastAnnotator software.

Although the number of assembled unigenes differs from species to species the overall distribution of sequences which are grouped in “molecular function”, biological process” and “cellular component” categories, level 2, are similar for all tested species (Figure 4B).

For example, in the “molecular function” classification the categories of “binding” and of “catalytic activity” are the largest one (respectively, 46% +/−2% and 33% +/−2%) in all species. In the “biological process” classification the first two represented categories are “cellular process” (25% +/−1.7%) and “metabolic process” (20% +/−2.5%). In the “cellular component” classification the first two represented categories in all species are “cell part” (45% +/−1.2%) and “macromolecular complex” (19% +/−1.2%).

These findings indicated that the Medfly 8–10****h MEET dataset contains, similar to other dipteran transcriptomes, a wide diversity of reconstructed transcripts from genes involved in a variety of conserved biological processes without any notable biases towards particular categories of genes. This result is expected, since early embryogenesis is highly conserved among dipterans. Differences between species are mostly due to temporal or spatial changes in gene expression [38], [82].

### Expression level analysis

To evaluate the relative expression level of the transcripts assembled in the MEET dataset we applied the RSEM software [83] using the FPKM metric (Fragments Per Kilobase of transcript per Million mapped reads) which normalizes paired-end read counts of transcripts by both their length and the total number of mapped reads in the sample [84]. The FPKM values were calculated using the Illumina reads generated from the single library we have produced.

We obtained a wide range of expression levels from less than 1 FPKM to about 200,000 FPKM (see Table S4). We clustered transcripts and unigenes according to their FPKM expression levels as reported in table 3. We considered those transcripts (37%) and unigenes (16%) as not expressed having a FPKM value<1. We observed that about 55% of transcripts have a low expression level (FPKM 1–10); about 8% have a moderate expression level (FPKM 10–100); and only 1.5% exhibit a high to extremely high expression level with values of >100 FPKM. As validation of this *in silico* expression analysis, qPCR was performed on total RNA extracted for the 8–10 hours AEL embryos to evaluate expression levels of 10 *C. capitata* genes and compared them to their respective FPKM values. We chose four housekeeping genes *superoxide dismutase* (*sod*), *ribosomal protein P1* (*rpP1*), *ribosomal protein S21* (*rps21*) and *glycerol-3-phosphate dehydrogenase* (*gpdh*), and six developmental genes (*tra-2*, *hopscotch* (*hop*), *outstretched* (*os*), *sisterless-A* (*sisA*), *deadpan* (*dpn*) and *virilizer* (*vir*) genes), known to be expressed during embryogenesis from previous studies in *Drosophila* and *C. capitata* [27], [55]. We normalized the respective expression levels obtained by the two methods and we found a high correlation (R = 0.93, Pearson; p-value of 3.29e-4) (see Methods; Table S5).

**Table 3:** FPKM distribution of the MEET data set.

FPKM accumulation level	N° of transcripts	% of transcripts	N° of genes	% of genes
0<FPKM<1, **very low**	15248	35,78	4221	16,04
1<FPKM<10, **low**	23162	54,35	18225	69,25
10<FPKM<10^∧^2 **moderate**	3557	8,35	3217	12,22
10^^∧^^2<FPKM<10^∧^3, **high**	529	1,24	542	2,06
10^∧^3<FPKM<10^∧^4, **very high**	110	0,26	108	0,41
FPKM>10^∧^4, **extremely high**	8	0,02	6	0,02
	**42614**	**100,00**	**26319**	**100,00**

To evaluate if the 647 transcripts with FPKM >100 have some enrichment in specific GO terms we performed a specific analysis. We compared their GO term frequencies for the molecular function category (LVL4) with the respect of those from the whole annotated MEET dataset (42,614 transcripts), using a custom R script (http://www.r-project.org/) which exploits the Fisher exact text. We considered in our analysis only the GO terms representing at least 1% of the total GO terms in our sample. We observed highly statistically supported enrichments (p-value 0.00001) for RNA binding (GO:0003723), hydrolase activity (GO:0016817) and unfolded protein binding (GO:0051082) (see Table S6). In contrast, the DNA binding (GO:0003677) term, often associated with transcription factors, was not enriched at this stage. The observation that RNA binding protein coding transcripts are highly expressed and overrepresented as GO term during this specific stage of embryogenesis is of particular interest considering that sex determination of insects is mostly based on sex-specific alternative splicing [85].

### Long Non Coding RNAs

In contrast to the increasing number of reports on the presence of long non-coding RNA (ncRNA) in human, mouse and other vertebrate systems, there is little knowledge about their distribution and function in invertebrates. A study in *Drosophila* revealed that a large proportion of intergenic regions encode ncRNAs and that particularly early stages of development express most abundant species of ncRNAs [86]. A recent study showed that in *Drosophila* many of these long ncRNAs are male-biased and preferentially associate with autosomes [87]. In the lepidopteran species *Bombyx mori*, a W-linked long non-coding RNA has been even implicated in acting as the primary signal in sex determination [57].

We performed a predictive analysis of coding potential of 18,337 non-annotated MEET transcripts (corresponding to 15,274 non-annotated unigenes) to search for putative long non-coding transcripts. We used three different applications: the Coding Potential Calculator (CPC, [88]), which uses machine-learning methods; the Portrait tool [89], which uses support vector machine and is optimized for non-model organisms; and the alignment-independent Coding Potential Assessment Tool, which uses logistic regression [90]. To ensure a high level of accuracy, we set a very stringent threshold for all three software outputs (see Methods and Table S7). In total 861 putative long non-coding assembled transcripts were identified, corresponding to 846 unigenes. A very similar number was recently reported in *Drosophila* (528; [87]). Interestingly, the GC content of these transcripts is 27.99%, significantly lower with respect to that of whole MEET data set (37.35%). Low GC content is one of the emerging feature of the long non-coding transcripts at least in human [91] and hence, this finding supports reliability of our prediction.

**Figure 5:**
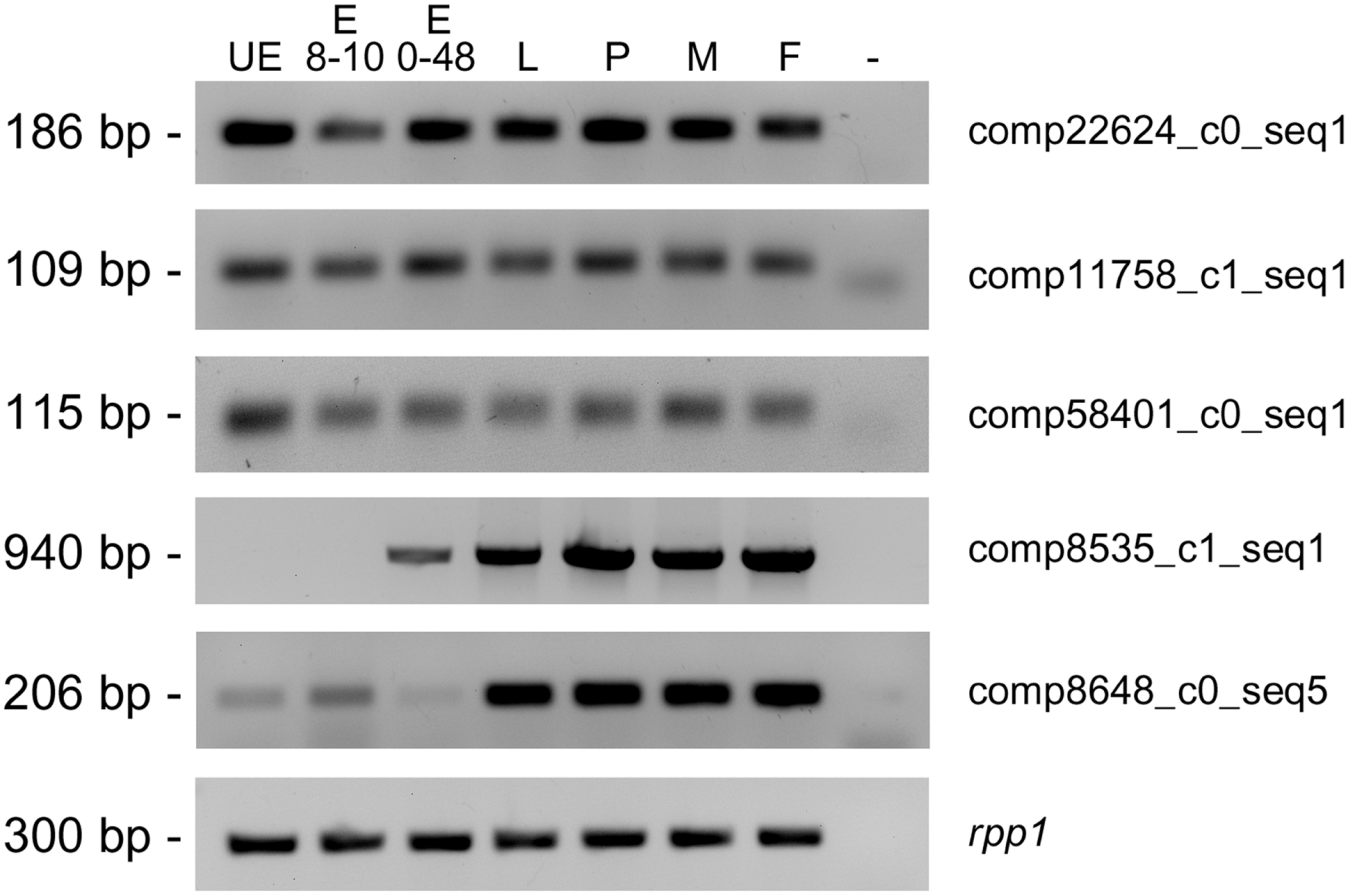
Temporal expression analysis of putative non-coding RNAs. UE = unfertilized eggs; E8–10****h = embryos at the stage of 8–10 hours after egg laying (AEL); E0–48****h = embryos at the stage of 0–48 hours AEL; L = third instar larvae; P = pupae; M = adult males; F = adult females. All the samples constituted individuals of both sexes.

To verify whether the predicted long ncRNAs were true transcripts and to exclude them if they were assembly artifacts we selected seven putative long ncRNAs between the more expressed ones (FPKM>25) and the longest one (length>800 bp) and we performed an RT-PCR analysis on RNA from embryos at the stage of 8–10 hours after egg laying. We further decided to monitor the expression profile of the seven putative ncRNAs during development of Medfly using total RNA from unfertilized eggs, embryos at the stage of 0–48 hours after egg laying, third instar larvae, pupae, and adult males and females. We obtained amplification products of the expected size for five out of seven ncRNAs, which were cloned and sequenced (Figure 5). Three of these were highly expressed (comp22624_c0_seq1, comp11758_c1_seq1 and comp58401_c0_seq1) and present in all developmental stages tested. Expression of the other two putative ncRNAs appears to be regulated during development. For instance, the expression of transcript comp8535_c1_seq1 starts at the late embryonic stage and continues until adulthood The transcript comp8648_c0_seq5 is initially present at low levels in UE and embryos, but undergoes a marked increase in expression levels from larval stage until adulthood (Figure 5). The five validated long ncRNAs were utilized as queries in BLASTN searches against *Drosophila* genome and NONCODE v4 database, a database of all kinds of non-coding RNAs (noncode.org). We could not retrieve any significant matches.

### Orthologs of *Drosophila* genes involved in sex determination

In *Drosophila*, female development is instructed by a double dose of the X chromosome. This signal activates a cascade of genes (*Sxl* > *tra*) which culminates in the female expression of the *doublesex (dsx)* gene at the bottom of the cascade. The presence of one X chromosome does not activate the cascade. As a consequence *dsx* is expressed in the male mode and male development follows. The sex determination pathway in *C. capitata* is only partially conserved with respect to that of *Drosophila* [15], [16]). While *tra* and *dsx* are structurally and functionally conserved it is not clear how *tra* is regulated in the Medfly. In particular, it is unclear how the male determining Y-linked M factor prevents activation of *tra*. Also, it is not known whether other *Drosophila* orthologs are involved as secondary players in the pathway [15], [16], [55].

To address these questions we searched for sex determining orthologs in the MEET dataset. We started with a list of 211 *Drosophila* genes from Flybase which include the key word “sex determination”. We filtered this initial set using “sex determination” as a GO term (GO:0007530) in Biological Process GO Class recovering a shortened list of 19 genes. We added to this list (Table 4), 6 more genes listed by Gomulski et al. (2008) as sex determining (see [27], table 2). We used the corresponding *Drosophila* proteins as queries to perform a TBLASTN analysis (with a cut-off value of 1e-10) against the MEET dataset. In case of genes encoding multiple isoforms, the protein having the longest ORF was selected. For the *tra* gene we used the *C. capitata* TRA protein as a query due to the very low level of similarity to the TRA protein of *Drosophila* [54]. Out of the initial set of 25 genes we identified *Ceratitis* orthologs for 20, including *tra*, *tra-2*, *Sex-lethal* (*Sxl*) and *intersex* (*ix*) genes (Table 4). Of the five genes, which were missing in our dataset, three genes are known not to be expressed at early embryonic stages in *Drosophila*: *dissatisfaction* (*dsf*); *hermaphrodite* (*her*); and CG3726.The *dsx* gene is expressed in *C. capitata* from 10****h after oviposition [55], [92]–[94]. Although *fru* gene is expressed throughout *Drosophila* embryogenesis producing various isoforms [95], we failed to identify FRU encoding transcripts in the MEET dataset, most likely due to its narrow and specific temporal window.

**Table 4:** Sex determination orthologs identified in the MEET data set.

Sex determination; Biological process; GO:0007530											
#	Dmsymbol	Dm name	Dmannotation id	meetBASEhortholog ID	Alignmentlenght (aa)	e-Value	Identity(%)	Similarity(%)	FPKM	FullORF	ORFlenght(aa)
1	da	daughterless	CG5102	comp8485_c1_seq2	157	5,00E-66	51	60	53,09	yes	712
2	dgrn	degringolade	CG10981	comp9048_c0_seq8	56	1,00E-15	57	69	14,74	yes	369
3	dpn	deadpan	CG8704	comp11618_c0_seq5	224	1,00E-78	66	77	11,73	no	226
4	dsf	dissatisfaction	CG9019	-	-	-	-	-	-	-	-
5	dsx	doublesex	CG11094	-	-	-	-	-	-	-	-
6	emc	extramacrochaetae	CG1007	comp9421_c1_seq4	168	3,00E-41	55	64	703,73	yes	242
7	fl(2)d	female lethal d	CG6315	comp12209_c0_seq13	200	3,00E-75	93	97	16,14	yes	652
8	fru	fruitless	CG14307	-	-	-	-	-	-	-	-
9	her	hermaphrodite	CG4694	-	-	-	-	-	-	-	-
10	hop	hopscotch	CG1594	comp9764_c0_seq2	1165	0.0	39	57	30,53	yes	1149
11	ix	intersex	CG13201	comp7792_c0_seq1	143	8,00E-57	72	86	82,39	yes	139
12	os (sisC)	outstretched	CG5993	comp11260_c0_seq3	308	6,00E-47	43	57	13,02	yes	411
13	sc (sisB)	scute	CG3827	comp3154_c0_seq1	73	2,00E-16	69	83	2,94	yes	282
14	sisA	sisterless A	CG1641	comp10723_c0_seq1	142	1,00E-11	28	53	175,56	yes	184
15	Stat92E	Signal-transducerand activatoroftranscriptionprotein at 92E	CG4257	comp10855_c0_seq2	770	0.0	46	62	118,69	yes	725
16	Sxl	Sex lethal	CG43770	comp11506_c0_seq8	278	9,00E-104	73	78	102,41	yes	338
17	tra	transformer*	CG16724	comp11901_c1_seq5	*	*	*	*	10,56	yes	429
18	tra2	transformer-2	CG10128	comp10827_c1_seq5	104	4,00E-34	67	87	272,94	yes	251
19	vir	virilizer	CG3496	comp11922_c0_seq8	973	0.0	62	76	12,38	yes	1432
20	gro	groucho	CG8384	comp9374_c0_seq1	733	0.0	89	91	223,55	yes	724
21	Mes-4	Mes-4	CG4976	comp8295_c0_seq1	477	0.0	65	79	4,22	yes	507
22	mod(mdg4)	modifier ofmdg4	CG32491	comp5246_c0_seq2	120	5,00E-64	89	96	356,86	yes	463
23	lolal	lola-like	CG5738	comp11328_c0_seq22	127	1,00E-72	100	100	166,07	yes	127
24	lola	Longitudinalslacking	CG12052	comp10714_c0_seq2	213	6,00E-74	71	76	545,70	yes	990
25	CG3726	-	CG3726	-	-	-	-	-	-	-	-

We found that practically all MEET reconstructed mRNAs (19 out of 20 orthologs) contained full length ORFs. All of the identified sex determination ortholog genes were expressed with an FPKM>1 with the top expressed genes corresponding to *extra macrochaetae* (*emc -* FPKM = 703.73), *longitudinals lacking* (*lola -* FPKM = 545.70), *modifier of mdg4* (*mod(mdg4)*) - FPKM = 356.86), *tra-2* (FPKM = 272.94), *groucho* (*gro -* FPKM = 223.55) and *sis-A* (FPKM = 175.56).

These top expressed sex determination genes encode DNA-binding proteins involved in transcriptional activity regulation except for the *tra-2* gene, which encodes an RNA-binding protein involved in alternative splicing regulation. As in *C. capitata* also in *Drosophila* the orthologs of *emc*, *lola*, *modg(mdg4)*, *groucho* and *sis-A* are expressed at relatively high levels during this specific embryonic period of time. Only the *tra-2* gene has much higher expression when in *C. capitata* respect to *Dmtra-2* at this cellularization stage. This is quite intriguing because we know that *Cctra-2,* differently to *Drosophila,* performs novel additional function in *Ceratitis* being required for the *Cctra* female-specific splicing and autoregulation [16].

In *Drosophila* the *sis-A* gene encodes a bZIP transcription factor (basic leucine zipper). *sis-A* is one of the 2 potent indicators of X chromosome dose (both X-linked, of course), together with *sis-B*, that are required for the female-specific early transcription of the *Sex-lethal* gene and the establishment of its female-specific splicing regulation, as well as, the female-specific repression of dosage compensation, required on the contrary in XY individuals. This gene is expressed in *Drosophila* only during embryogenesis and its functions are linked to 1) the female-specific activation of *Sxl,* as well as 2) to endoderm migration and midgut formation in both sexes [96]. This second function is vital for both sexes while the first function is vital only for female XX embryos. In *C. capitata*, as in many other non Drosophilidae species, the ortholog of *Sxl* is not sex-specifically regulated and the encoded protein product is present in embryos of both sexes (Saccone et al., 1998). *Ceratitis* SIS-A shows 26% aa identity and 49% similarity to the *Drosophila* SIS-A and, as in *Drosophila*, it is expressed during embryogenesis but not at adult stages. Most likely the *C. capitata* ortholog is still required in both sexes, for the vital function of midgut formation, but not for female-specific *CcSxl* regulation, which most likely evolved only before or during Drosophilidae radiation [97].

Drosophila *lola* gene has multiple key functions during development of the nervous system of embryos/larvae until adulthood in both sexes and it seems to be involved indirectly to control the number of male-specific sex combs required for proper mating [98]. Furthermore, Lola protein shares with FRU the same BTB domain conferring DNA-binding abilities to both proteins. In *C. capitata lola* is also expressed at high levels during embryogenesis as well as in adults of both sexes, with very complex pattern of alternatively spliced isoforms (data not shown).

*Extra macrochaetae* participates in sensory organ patterning by antagonizing the neurogenic activity of the *achaete-scute* complex (AS-C), which includes *sis-B* (*sc*) involved also in early female sex determination. EMC lacks a basic DNA-binding domain but is able to form heterodimers with other HLH proteins, such as SIS-B. Both *sis-B* and *emc* seem to be involved in the X:A counting mechanism composing the *Drosophila* sex determination primary signal, respectively as X-linked numerator and denominator. This model has been recently challenged by a new one based only on X-linked elements (XSE; [99]. It is difficult to imagine that the *emc* ortholog in *C. capitata* and/or *sis-B* could play a role in sex determination, being in *Drosophila* upstream of *Sxl*, a gene not required for sex determination in non Drosophilidae species.

*Drosophila gro* transcripts are expressed at high levels in 2–6 hr embryos, and in adult females. In *C. capitata gro* is also expressed at high levels in embryos and preliminary data suggest that is expressed also in adult flies but is female-biased (6 folds; unpub. data).

## Conclusions

We produced a *de novo* transcriptome assembly of early *C. capitata* embryos (MEET; Medfly Early Embryonic Transcriptome) and identified 26,319 unigenes that seem to represent accurately the complexity present in 8–10 hours AEL embryos. Furthermore, the reconstructed transcripts seem to be not far off from their real full length *in vivo*, as suggested by three different analyses. We compared the sequence complexity of MEET and of reassembled *C. capitata* ESTs (from embryos, adults and tissues), concluding that we have identified ∼20,400 novel *C. capitata* unigenes (78%) 11,045 of which correspond to putative protein coding genes. The MEET contains, similarly to other dipteran transcriptome datasets, a broad diversity of transcripts from genes involved in a variety of different biological processes without any notable biases towards specific categories of genes. We identified 861 putative long ncRNAs and we validated their prediction on seven selected transcripts. We have also selected twenty *C. capitata* embryonic unigenes from the MEET, which are orthologs of *Drosophila* genes involved in sex determination (either directly or indirectly). We found that 19 out of 20 orthologs contained full length ORF. This *de novo* assembled transcriptome dataset is valuable tools for a wide range of different applications: developmental expression analyses, reverse genetic studies by RNAi, TALENs and CRISPR/cas-9, and improving transgenic strategies to produce male-only progeny. The MEET dataset will also be useful for the embryonic gene annotation in the on going Medfly Genome Project (Handler, A., USDA, pers. comm.).

## Methods

### Medfly rearing

*C. capitata* Benakeion adult flies were reared in standard laboratory conditions at 25°C, 70% relative humidity and 12∶12 h light-dark regimen. Flies were fed with yeast/sucrose powder (1∶2). A very large cross between about 800 males and 1600 virgin females was produced to obtain a high embryonic oviposition rate per hour. Embryos were collected in transparent plastic boxes filled with distilled water for two hour-long intervals. The collected embryos were allowed to age at 25°C for eight hours to get the synchronized embryonic sample of 8–10 hours after oviposition. Embryos were collected and transferred into a GIT extraction buffer (Guanidine isothiocyanate 4****M, EDTA 5****mM, NaAc 0.1****M, Sarkosyl 0.5%) and stored at −20°C.

### RNA extraction, Illumina paired-end cDNA library construction and sequencing

We produced a cDNA library for the 8–10****h embryonic sample using the NEB-Next Ultra RNA Library Prep Kit (New England Biolabs, USA) according to the manufacturer’s instructions. Briefly, high quality total RNA was extracted in GIT buffer and purified by CsCl gradient ultracentrifugation method. The integrity and purity of total RNA were assessed using Agilent Bioanalyzer, ThermoScientific NanoDrop and gel electrophoresis analyses. Poly-A+ containing mRNA was extracted from 2 µg of total RNA using oligo(dT) magnetic beads and fragmented for 2 minutes into 200–500 bp pieces using an ultrasonicator (Covaris, USA). The cleaved RNA fragments were copied into first strand cDNA using reverse transcriptase and random primers. After second strand cDNA synthesis, fragments were end repaired, a-tailed and Illumina adapters were ligated. The products were purified using AMPure XP beads (Beckman Coulter) and enriched by running PCR for 10 cycles to create the final cDNA sequencing library. The cDNA library was used for 100 bp paired-end (PE) sequencing on an Illumina Genome Analyzer IIx (Otogenetics Corporation, USA) producing 20,924,960 PE reads.

### *De novo* transcriptome assembly

The PE reads were *de novo* assembled using Trinity (release 2012-10-05) [65] on the ALAN Server at the Department of Biology, University of Naples Federico II (24 cores, 192 GB of memory). Trinity was run on the PE reads (raw or filtered) with the fixed default k-mer size of 25, minimum contig length of 200, maximum length expected between fragment pairs of 500, 22 CPUs, and a butterfly HeapSpace of 20 GB. We wished to verify if a filtering step could significantly improve the quality of our assembly, in terms of N50 value and average length of assembled transcripts, and hence we tested four filtering conditions with different stringency: n°1 (filtering condition named “trim”) trimming of 5 bp at 5′ and 3′ ends (where the lowest quality bases are usually located) of each read with Galaxy on-line tool [69], n°2 (named “trimmo”) quality control by sliding window analysis and adapter contamination removal by Trimmomatic software [68], n°3 (named “qc”) quality control on whole read length and adapter contamination removal with NGS-QC-Toolkit software [67] and n°4 (named “trim+qc”) trimming as for point 1 plus quality control and adapter contamination removal with NGS-QC-Toolkit software (see Methods S1). We produced four different Trinity assemblies and compared them with the assembly obtained by Trinity using raw reads. We took into account the following parameters: number of base pairs of reads utilized in the assembly (URbp), N50 value (N50), average transcript length (AVL), number of assembled transcripts (NoTr) and total number of base pairs of the assembled transcripts (ASbp). The rationale of our test is that if one of the filtering condition improves the quality of the assembly, we expect to obtain a reduction in URbp, ASbp and NoTR values but a significant improvement of N50 and AVL parameters. As expected, the URbp value decreases (from 10% to 48%) in the four filtered assemblies respect to the URbp of the assembly with raw data as well as the NoTr value (from 8% to 20%) (see Figure S1). The ASbp value instead decreases for all the filtering condition except for the “trimmo” condition where we observed a reduction of only 1%. Conversely, the N50 and the AVL values increase for the “trimmo” assembly (5.3% and 6.2% respectively). Based on this result we decided to use for our *de novo* transcriptome assembly the reads filtered with the “trimmo” condition.

Prior to the assembly step we depleted the filtered reads matching with the Medfly mitochondrial DNA sequence (GenBank acc. num.: AJ242872.1) and with the ribosomal RNA gene sequences (Cc18S, 5.8S, 16S and 28S; GenBank acc. num.: AF096450.2; AF189691; AY830884.1; KC177754.1) using the Bowtie aligner with zero mismatch allowed (-v 0 parameter) [70]. Assembled transcripts were blasted against NCBI UniVec database (http://www.ncbi.nlm.nih.gov/VecScreen/UniVec.html) to identify segments with adapter contamination. Human and bacterial sequence contamination was investigated using the web-based version of DeconSeq [71], with a query coverage and a sequence identity threshold of 90%.

### Functional annotation

The assembled transcripts were annotated applying the FastAnnotator annotation pipeline with default parameters [72] to investigate and summarize their functional categories. The annotation in FastAnnotator includes four main parts: finding best hits in the NCBI nr database (December 2012 update) using LAST alignment algorithm, assigning GO terms, identifying enzymes, and identifying domains. FastAnnotator runs these four steps in parallel to speed up the annotation procedure.

To perform the comparative analysis of the MEET GO terms distribution with other dipteran high-throughput embryonic transcriptomic data, we downloaded SRA file for the following species: *D. melanogaster* (2–16****h old embryos) (SRR042295 and SRR058885); *B. dorsalis* (0–24****h old embryos) (SRR316210); *C. albipunctata* (8–12****h old embryos) (ERR160071); *M. abdita* (0–4****h old embryos) (ERR196167); *E. balteatus* (3–6****h old embryos) (SRR190625). The raw data were assembled without any filtering step and with default parameters using Trinity. Functional annotations were performed for each species, applying the FastAnnotator pipeline with default parameters.

### Evaluation of coding potential

The prediction of coding potential of transcripts not annotated in our MEET data set was performed using three independent different prediction methods: the Coding Potential Calculator [88], the Portrait software [89] and the Coding Potential Assessment Tool [90]. We applied available web-tools in CPC and Portrait, while the CPAT software was installed locally (v1.2.1) using *D. melanogaster* training data set as a reference. In CPC a positive coding potential score indicates a protein coding potential of the respective target transcript, whereas negative values predict non-coding potential of transcripts. In Portrait the coding and non-coding potential of transcripts are expressed in percentage while in CPAT the coding probability score ranges between 0 and 1 with coding probability cut-off usually set at ≥0.5. In order to extract potential non-coding transcripts with a high reliability from our dataset, we selected very stringent thresholds for the three prediction methods as following: CPC coding potential score <−1.2, Portrait non-coding probability >95% and CPAT coding probability <0.01. Only those transcripts in accordance, at the same time, with the three very conservative cut-offs were selected as putative early embryonic non-coding transcripts.

### Expression analysis and Real Time-PCR validation

We applied the RSEM software and the BOWTIE aligner, as implemented in the Trinity software package, to assign reads to genes and isoforms and to compute transcript expression levels using FPKM metric. Transcripts with FPKM value above 100 were further investigated for Gene Ontology (GO) enrichment using the R language.

We validated expression levels of ten selected genes by Real-Time qPCR. We prepared cDNA using 50 ng of the polyA+ RNA utilized for the sequencing library preparation in a 60 µl reaction using the EuroScript M-MLV Reverse Transcriptase (Euroclone, ITALY) following the manufacturer’s instructions. The primers used in qPCR reactions were designed using the Primer Express software (Applied Biosystems, USA) and are listed in Methods S2. qPCR was performed in triplicate with 1 µl of a 1∶5 cDNA dilution for each target gene using Brilliant III Ultra-Fast SYBR Green qPCR Master Mix (Agilent Technologies, USA) and a 7500 Real-Time PCR System (Applied Biosystems, USA). Cycling parameters were: 2 min at 50°C, 10 min at 95°C, 40 cycles of 15 sec at 95°C and 60 sec at 60°C, 15 sec at 95°C, 1 min at 60°C, 30 sec at 95°C, 15 sec at 60°C. We applied the R_n_ method (R_n_ = R_o_ of the target gene/R_o_ of the normalizator gene) for relative gene expression analysis and the Real-Time PCR Miner tool [100] using the Medfly *superoxide dismutase* gene (GenBank acc. num.: L35494.1) as normalizator gene. We then compared, by performing a Pearson Correlation test in the R language, the R_n_ values of the nine selected genes with their normalized FPKM values (obtained by dividing the FPKM of the target gene by the FPKM value of the SOD gene).

### RT-PCR validation of non-coding RNAs

Total RNA was extracted using TRIzol (Life Technologies, USA) from adult individuals and from unfertilized eggs, embryos, larvae and pupae of *C. capitata*. For the 8–10****h old embryonic sample the same total RNA utilized for the sequencing library preparation was used. Oligo-dT-primed cDNA was prepared from 1 µg of DNAse I-treated total RNA using the EuroScript M-MLV Reverse Transcriptase (Euroclone, ITALY) following the manufacturer’s instructions. 1/20 v/v of the synthesised cDNA was amplified by PCR at non-saturating condition as described in Salvemini et al., (2006) [101]. RT-PCR products were analysed by agarose gel electrophoresis. Primers utilized in RT-PCR developmental expression analysis are listed in Methods S2.

## Supporting Information

Figure S1 **Filtering condition test for the Trinity** ***de novo*** **assembly of MEET data set.** Comparison of the five assemblies obtained with raw data and the four different filtering conditions (trim, trimmo, qc and trim+qc). For each assembly: the blue line indicates the number of base pairs of Reads Utilized (URbp); the red line indicates the N50 values; the green line indicates the average transcript length (AVG); the purple line indicates the total Number of assembled TRanscripts (NoTR); the azure line indicates total number of base pairs of the ASsembled transcripts (ASbp). Values for the five parameters were arbitrarily corrected with correction factors indicated in the figure to obtain a clearer comparative graph.
(TIF)

Table S1 **Comparison with** ***C. capitata*** **full-length cDNAs.**
(XLS)

Table S2 **OHR and FLHR analyses.**
(XLS)

Table S3 **ESTs assembly statistics.**
(XLS)

Table S4 **FPKM values of all assembled transcripts.**
(XLS)

Table S5 **Comparison of RNA-seq and qPCR expression levels.**
(XLS)

Table S6 **GO enrichment analysis.**
(XLS)

Table S7 **Non-coding RNA prediction.**
(XLS)

Methods S1 **List of software utilized for the four tested filtering conditions.**
(DOC)

Methods S2 **List of primers utilized in this study.**
(DOC)
