# Supplementary figures and images for: De Novo Assembly and Transcriptome Analysis of the Mediterranean Fruit Fly Ceratitis capitata Early Embryos
Source: PLoS One. 2014 Dec 4;9(12):e114191. doi: 10.1371/journal.pone.0114191 (PMC4256415; doi:10.1371/journal.pone.0114191)

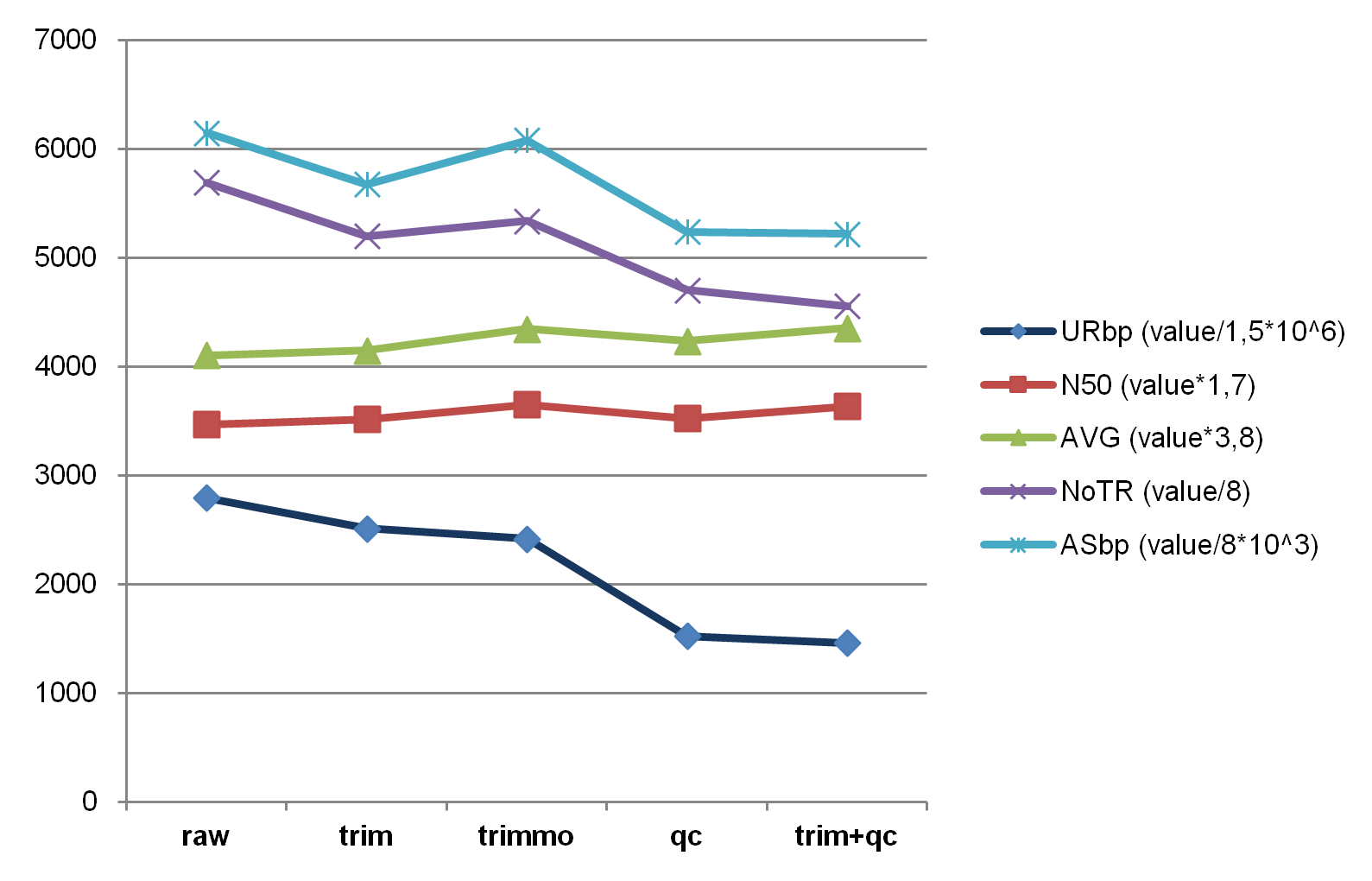

Supplement: Figure S1 — Filtering condition test for the Trinity de novo assembly of MEET data set. Comparison of the five assemblies obtained with raw data and the four different filtering conditions (trim, trimmo, qc and trim+qc). For each assembly: the blue line indicates the number of base pairs of Reads Utilized (URbp); the red line indicates the N50 values; the green line indicates the average transcript length (AVG); the purple line indicates the total Number of assembled TRanscripts (NoTR); the azure line indicates total number of base pairs of the ASsembled transcripts (ASbp). Values for the five parameters were arbitrarily corrected with correction factors indicated in the figure to obtain a clearer comparative graph. (TIF) [file pone.0114191.s001.tif]
