# Supplementary material for: De Novo Assembly and Transcriptome Analysis of the Mediterranean Fruit Fly Ceratitis capitata Early Embryos
Source: PLoS One. 2014 Dec 4;9(12):e114191. doi: 10.1371/journal.pone.0114191 (PMC4256415; doi:10.1371/journal.pone.0114191)
Supplement: Methods S1 — List of software utilized for the four tested filtering conditions. (DOC) [file pone.0114191.s009.doc]

**Methods S1 - Software utilized for the four tested filtering conditions**

**1) trim**

Trimming of 5 bp at 5' and 3' ends (where the lowest quality bases are usually located) of each read with Galaxy on-line tool (Blankenberg et al., 2010):

This tool is based on FASTX-toolkit by Assaf Gordon.

<https://usegalaxy.org/>

NGS TOOLBOX BETA

NGS: QC and manipulation

Trim sequences

**2) trimmo**

Quality control by sliding window analysis and adapter contamination removal by Trimmomatic software (Lohse et al. 2012) with the following parameters and Illumina adapter sequences:

trimmomatic-0.25.jar org.usadellab.trimmomatic.TrimmomaticPE -threads 22 -trimlog trimmomatic.log 1.fastq 2.fastq 1_trim_pair.fastq 1_trim_unpair.fastq 2_trim_pair.fastq 2_trim_unpair.fastq ILLUMINACLIP:illumina_adapters.fa:2:40:15 LEADING:3 TRAILING:3 SLIDINGWINDOW:4:15 MINLEN:50

Using Clipping Sequence: 'AATGATACGGCGACCACCGAGATCTACACTCTTTCCCTACACGACGCTCTTCCGATCT'

Using Clipping Sequence:

'ACACTCTTTCCCTACACGACGCTCTTCCGATCT'

Using Clipping Sequence:

'GATCGGAAGAGCGGTTCAGCAGGAATGCCGAG'

Using Clipping Sequence:

'CGGTCTCGGCATTCCTGCTGAACCGCTCTTCCGATCT'

Using Clipping Sequence:

'ACACTCTTTCCCTACACGACGCTCTTCCGATCT'

Using Clipping Sequence: 'CAAGCAGAAGACGGCATACGAGATCGGTCTCGGCATTCCTGCTGAACCGCTCTTCCGATCT'

**3) qc**

Quality control on whole read length and adapter contamination removal with NGS-QC-Toolkit software (Patel and Jain, 2012) and parameters –l 80 (the cut-off value for percentage of read length that should be of given quality) and –s 30 (the cut-off value for PHRED quality score for high-quality filtering).

**4) trim+qc**

Trimming as for point 1 plus quality control and adapter contamination removal with NGS-QC-Toolkit software with the same parameters of point 3.
