# Supplementary material for: De Novo Assembly and Transcriptome Analysis of the Mediterranean Fruit Fly Ceratitis capitata Early Embryos
Source: PLoS One. 2014 Dec 4;9(12):e114191. doi: 10.1371/journal.pone.0114191 (PMC4256415; doi:10.1371/journal.pone.0114191)
Supplement: Methods S2 — List of primers utilized in this study. (DOC) [file pone.0114191.s010.doc]

**Methods S2 - List of primers utilized in this study**

**RT-PCR of putative long non-coding transcripts**

7740+: 5'-CGCAGAGTACATGGGGAGTT

7740-: 5'-CAACAAAACCGCATAGCCAGT

8535+: 5'-ACTGTTGGTTCTGCTGCAAAT

8535-: 5'-TTGCTTACGCTCTTTCACTTGC

8648+: 5'-CATTTCCCCCATGAGCCTGA

8648-: 5'-GCGAAGGCGTTTATCACAAGG

10110+: 5'-GGACGGACGTGTGTGTTTTG

10110-: 5'-GCAAGGAACGGTTAATGGCG

11758+: 5'-GCAAATAGGTGCAGGAAACTT

11758-: 5'-GTGTACGCGTAATTAGCACAA

22624+: 5'-TGGAGCTCACAAACATGCCA

22624-: 5'-CAAAGCGCAAAGCAAATGGG

58401+: 5'-GCAACGGTGAAAAGAAACGA

58401-: 5'-CCTAATGTGAGTGAGTGTGT

**qRT-PCR**

deadpan+: 5'-ATTCGGACGACGATTTCGAT

deadpan-: 5'-AAGCCGTGTGGATTCGACAT

hopscotch+: 5'-GAAAACCATCGATCTGAGTACCATAA

hopscotch-: 5'-AACTGCGCCTTTAGAAATAAATCC

outstretched+: 5'-GTTTATTTGTTTGTGCGTGTGTTTG

outstretched-: 5'-CCATTGACGAGTGAACAGTATCG

sisterlessA+: 5'-TGCATCCTGCCGTAAATCAC

sisterlessA-: 5'-CCTCTCTGCTTCCTGCAGTTG

virilizer+: 5'-GAAGGCATACGTTATTTCCTAATTGG

virilizer-: 5'-AGAGCGAATTTGGTACGTGTGA

tra-2gen+: 5'-ACCGTTGTATAGGAGTTTTTGGT

tra-2gen-: 5'-ACTACTTGTATCCTCTCGATTGGT

qRPP1+: 5'-GGCTTTGGAAGGTATCAACGTT

qRPP1-: 5'-TCTGGTTCTTCCTCCTTCTTCTTCT

qGPDH+: 5'-CCAATTTGGCCAACGAAGTG

qGPDH-: 5'-ACAAACTTCAACGGCATCAGAGT

qRPS21+: 5'-TCCACGCTAAGGATCATGCTT

qRPS21-: 5'-TGGCCAAACGCACAATACAA

qSOD+: 5'-GCTGCTCCGAGAACGTTCA

qSOD-: 5'-GTGCCCCGTGATCCATCTTA
